# Autonomic dysfunction as manifestation of ICANS: A case report

**DOI:** 10.1097/MD.0000000000038659

**Published:** 2024-09-06

**Authors:** Dina Rochate, Antonio Manuel González-García, Carmen Santos Marcos, Estefanía Pérez-López, Ana-África Martín-López, Mónica Alaña, Lourdes Martín Martín, Miriam López Parra, Fermín Sánchez Guijo, Lucía López Corral

**Affiliations:** a Hematology Department, Hospital do Divino Espírito Santo de Ponta Delgada, Ponta Delgada, Portugal; b Neurology Department, University Hospital of Salamanca, Salamanca, Spain; c Hematology Department, University Hospital of Salamanca-IBSAL, Centro de Investigación del Cáncer, Salamanca, Spain; d Flow Cytometry Service, University Hospital of Salamanca-IBSAL, Centro de Investigación del Cáncer, Spain.

**Keywords:** chimeric antigen receptor T cells-dysautonomia, diffuse large B-cell lymphoma, ICANS, neurotoxicity, tachycardia

## Abstract

**Rationale:**

Anti-CD19 chimeric antigen receptor T-cell (CAR-T) therapy is a successful treatment for B-cell malignancies associated with cytokine release syndrome (CRS) and immune effector cell-associated neurotoxicity syndrome (ICANS). Cardiovascular toxicities have also been reported in this setting. However, there is scarce data regarding development of autonomic disorders after CAR-T cell therapy.

**Patient concerns:**

We report a case with a patient with non-Hodgkin B-cell lymphoma, refractory to 2 prior lines of immunochemotherapy, treated with CAR-T therapy.

**Diagnoses:**

Orthostatic hypotension secondary to autonomic dysfunction was diagnosed as manifestation of ICANS.

**Interventions:**

The patient received metilprednisolone 1000 mg IV daily for 3 days and anakinra 100 mg IV every 6h.

**Outcomes:**

The vast majority of autonomic symptoms ceased and 4 months after CAR-T therapy, autonomic dysfunction was resolved.

**Lessons:**

New-onset autonomic dysfunction can occur as manifestation of ICANS in patients who experience persistent neurologic and cardiovascular symptoms after resolution of acute neurotoxicity and should be early recognized. Differences in differential diagnosis, mechanisms and treatment approaches are discussed.

## 1. Introduction

Anti-CD19 chimeric antigen receptor T-cell (CAR-T) therapy is a successful treatment for B-cell malignancies, though its association with multiple adverse effects, including cytokine release syndrome (CRS) and immune effector cell-associated neurotoxicity syndrome (ICANS), is well described.^[1]^ Due to its potentially life-threatening nature, ICANS is considered a severe complication of CAR-T therapy, which occurs in 20% to 60% of patients, of whom 12% to 30% have severe (≥grade 3) symptoms.^[2]^ Its pathophysiology remains to be elucidated but it is thought to be secondary to inflammatory cytokines action related to disruption of blood-brain barrier (BBB) due to either its passive diffusion into the brain or the presence of activated T-cells in the central nervous system (CNS).^[3,4]^ Symptoms of ICANS are variable and can initially be vague. Typically, ICANS manifests as frontal encephalopathy, however, its clinical spectrum can highly vary in type and severity, ranging from a mild headache to severe impairment including delirium, seizures or cerebral edema.^[5]^ ICANS is a clinical diagnosis and brain magnetic resonance imaging (MRI) and cerebrospinal fluid (CSF) evaluation are necessary to rule out alternative diagnosis. Management of ICANS is based on the severity of the score and the concurrence of CRS.^[4]^ The prognosis is good and the majority of patients fully recover without any long-term sequelae. Cardiovascular side effects related to CRS were recently described, including cardiovascular mortality, new onset heart failure, heart failure decompensation, and new arrhythmias. Although arterial hypotension and orthostatic hypotension were studied as effects of CAR-T therapy, autonomic disorders have been described but not clearly characterized as ICANS manifestations.^[6]^ Postural orthostatic tachycardia syndrome (POTS), is a heterogeneous multifactorial disorder characterized by orthostatic tachycardia with associated symptoms and relief by recumbent posture and accompanied by evidence of autonomic dysfunction.^[7]^ Potential causes of orthostatic hypotension have been suggested, such as older age, history of hypertension and use of medications (anti-hypertensives, diuretics, antidepressants and neuroleptics). Orthostatic hypotension may be due to autonomic nervous system dysfunction, with different clinical manifestations depending on whether sympathetic or parasympathetic involvement predominates.^[8]^ In neuropathies, there may be autonomic involvement, with different cardiovascular symptoms. There is also a relationship between dysautonomia and various inflammatory processes. There is a growing body of evidence that the etiology of POTS (one of the most common autonomic disorder, with a wide range of clinical manifestations, such as postural tachycardia, orthostatic intolerance, presyncope, dyspnea, chest pain, exercise intolerance and noncardiac manifestations such as dizziness, headaches, muscles weakness, fatigue and sleep disturbances),^[9]^ may have immune-mediated pathogenesis as it was recently portrayed with association with COVID-19.^[7, 10]^ Also, a case of dysautonomia as a manifestation of neurotoxicity after CAR-T therapy has been described in literature.^[3]^ Considering our limited understanding of autonomic dysfunction after CAR-T therapy, we report a case of a patient with non-Hodgkin-B-cell lymphoma treated with anti-CD19 CAR-T therapy who developed orthostatic hypotension secondary as manifestation of ICANS.

## 2. Case presentation

**Figure 1.**
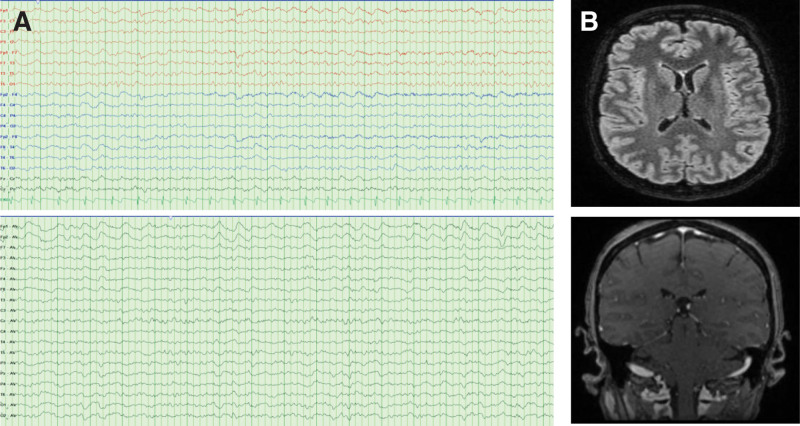
(A) Anterior-posterior bipolar montage and referential montage EEG with intermittent bursts of bilateral frontal intermittent rhythmic delta activity (FIRDA) over an underlying mild diffuse slowing of background activity; (B) FLAIR-T2 weighted axial view and T1-weighted dynamic contrast-enhanced coronal view brain MRI with normal results. EEG = electroencephalogram, MRI = magnetic resonance imaging.

**Figure 2.**
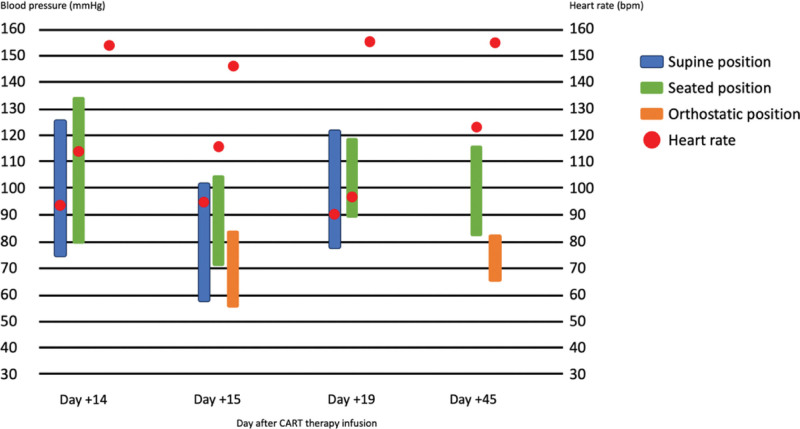
Graphic evolution of blood pressure and heart rate measurements. X-axis - days after CAR-T therapy infusion; Y-axis—blood pressure measured in mm Hg; blue represents blood pressure measured in supine position; green represents blood pressure measured in a seated position; orange represents blood pressure measured in supine position; red represents heart rate during measurements in bpm.

**Figure 3.**
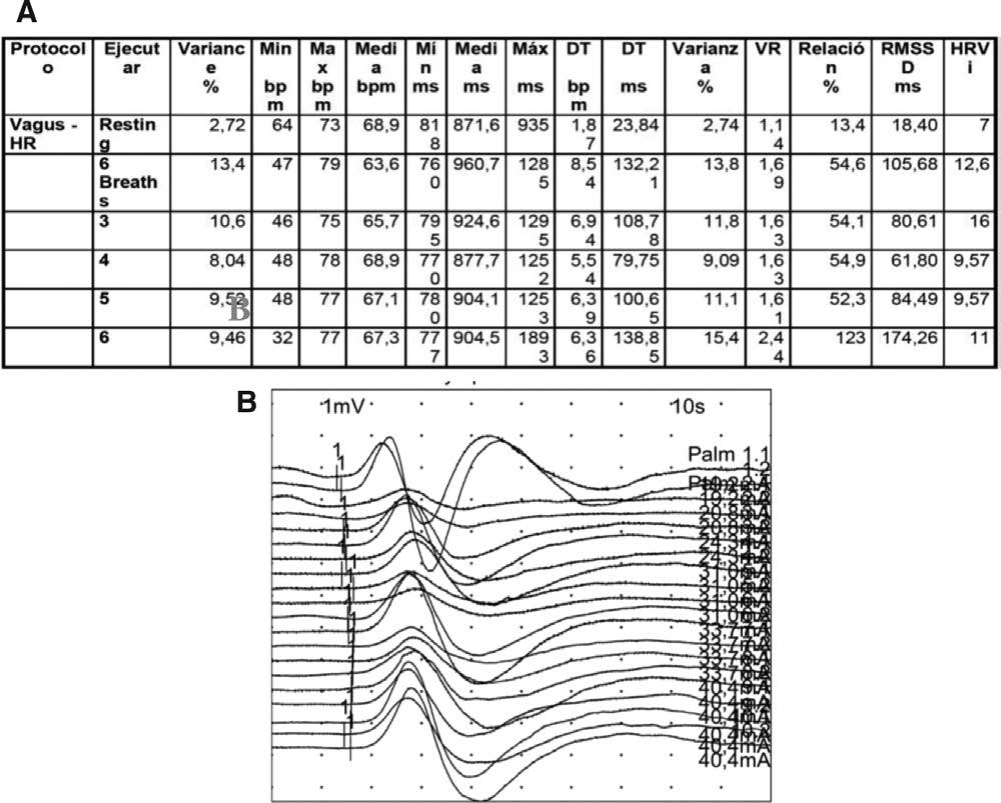
(A) R-R Interval Analysis. Basal heart rate (73 bpm) increases after 5 min of breathing at 6 cycles per minute (79 bpm). (B) Sympathetic skin response with normal results.

**Figure 4.**
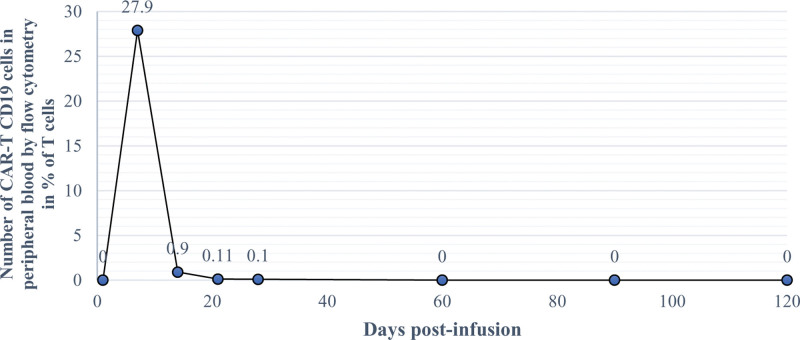
Graphic with evolution of peripheral blood (PB) CAR-T cells by flow cytometry: X axis—days after CAR-T therapy infusion (+1, +7, +14, +21, +28, +60, +90, and + 120 d); Y axis—number of CAR-T CD19 cells in PB by flow cytometry in %.

A 46-year-old woman with no previous medical history was diagnosed with primary mediastinal large B-cell lymphoma (PMBCL), with bulky mediastinic mass (17 × 13 × 12 cm) stage IIA-X, IPI-1. The patient had been primary refractory to 2 prior lines of immunochemotherapy: dose-adjusted etoposide, prednisone, vincristine, cyclophosphamide, doxorubicin, and rituximab and polatuzumab, vedotin plus rituximab, ifosfamide, carboplatin and etoposide (Pola-R-ICE). Given the refractoriness of the disease, the patient was considered candidate for anti-CD19 CAR T-cell axicabtagene ciloleucel (Axi-Cel). After lymphocyte apheresis the patient was in disease progression and no bridging therapy was given before axicabtagene ciloleucel (Axi-Cel). At the time of lymphodepleting chemotherapy (LDC), the patient had an ECOG 0, and her analytic study showed grade 2 neutropenia, adequate renal and liver function and normal lactate dehydrogenase. CAR-HEMATOTOX score was 0 (low-risk group). She received 3 consecutive days of fludarabine (30 mg/m^2^) and cyclophosphamide (500 mg/m^2^) as LDC and a target dosage of 2.0 × 106/kg body weight of viable anti-CD19 CAR-positive T cells was infused. The patient developed early grade 1 CRS on day + 1 (at 24h after infusion) and it was managed with supportive care. As the patient presented persistent CRS despite supportive measures, she was treated with 2 administrations of tocilizumab 8 mg/kg at 48 hours. The second dose of tocilizumab and a single dose of dexamethasone 10 mg were required due to fever persistence for 48 hours on day + 5, with resolution. On the same day, despite CRS resolving, the patient showed inattention, bradypsychia and upper limb tremors. Immune effector cell-associated encephalopathy (ICE) scale was performed, and the patient made writing and attention mistakes (ICE 8/10 points). ICANS grade 1 diagnosis was stablished and an urgent brain CT was performed, with normal results. Treatment with dexamethasone 10 mg IV every 6 hours was initiated. Nevertheless, the patient developed a rapid worsening, altered mental status with global aphasia, Glasgow Coma Scale punctuation was 10 (spontaneous eye opening, no verbal response and localizing pain) and the electroencephalogram (EEG) revealed intermittent bursts of bilateral frontal intermittent rhythmic delta activity (FIRDA) and signs of mild encephalopathy (Fig. 1–A), consistent with grade 3 ICANS. Given the need for more frequent monitoring of her neurological status, the patient was admitted to intensive care unit and received methylprednisolone 1000 mg IV daily for 3 days followed by a taper to discontinuation over 1 week and anakinra 100 mg IV every 6h. Seizure prophylaxis was provided with levetiracetam (500 mg twice a day) before CAR-T cell infusion, nonetheless per the guidance of a neurology consultation, levetiracetam was increased to 1000 mg twice a day. The CSF study and brain MRI showed no alterations (Fig. 1B) and infectious studies were negative. CSF flow cytometry detected 66.5% of CAR-T cells, with minimal residual disease negative. When 4 doses of anakinra were administered, a consistent neurological improvement was observed (ICANS grade 1) and anakinra was tapered over an 8-day course without neurological worsening. Scarcely ICANS was solved, on day + 14, in discontinuation of corticosteroids and no longer receiving anakinra, the patient suffered gait disturbance and drug-resistant nausea and vomiting, initially dependent on head movements, but subsequently appeared to be related to orthostatic position. Despite normal neurological examination and utter resolution of ICANS, the symptoms persisted. Blood pressure was measured in supine and upright positions, confirming orthostatic hypotension and tachycardia (Table 1, Fig. 2), with symptomatic pre-syncope. Control supplementary testing was performed (brain MRI and EEG), with normal results. After ruling out adrenal insufficiency and pharmacological causes, orthostatic hypotension due to autonomic dysfunction was suspected. Gradually, the vast majority of autonomic symptoms ceased, leading to no requirement for treatment with fludrocortisone. The patient was encouraged preventive treatment with postural measures. The patient was discharged after 20 days since CAR-T therapy infusion, having solely occasional dizziness with orthostatism. Three months after neurotoxicity onset neurophysiological studies were performed such as sympathetic skin response and R-R interval analysis, and they showed cardiovagal autonomic impairment (parasympathetic dysfunction) with normal sympathetic system function (Fig. 3A and B). Therefore, the patient was diagnosed with orthostatic hypotension secondary to autonomic dysfunction as manifestation of ICANS. The detection of CAR-T cells in PB is presented in Figure 4. Four months after CAR-T cell infusion, autonomic dysfunction was resolved. However, disease progression was confirmed by positron emission tomography scan. The patient was proposed on pembrolizumab (200 mg/every 3 weeks) and 5 months after CAR-T therapy achieved partial response.

**Table 1:** Evolution of blood pressure and heart rate measurements.

D + 14	Supine position	Seated position	Orthostatic position
Systolic blood pressure (mm Hg)	124	133	NM*
Diastolic blood pressure (mm Hg)	77	79	NM*
Heart rate (bpm)	94	113	154

D + 15	Supine position	Seated position	Orthostatic position
Systolic blood pressure (mm Hg)	101	103	NM*
Diastolic blood pressure (mm Hg)	58	71	NM*
Heart rate (bpm)	95	116	147*

D + 19	Supine position	Seated position	Orthostatic position
Systolic blood pressure (mm Hg)	121	119	82
Diastolic blood pressure (mm Hg)	78	89	57
Heart rate (bpm)	90	96	157

D + 45	Supine position	Seated position	Orthostatic position
Systolic blood pressure (mm Hg)	NM**	115	83
Diastolic blood pressure (mm Hg)	NM**	82	67
Heart rate (bpm)	NM**	123	157

NM* - non-measurable > 2 attempts with pre-syncope symptom; NM** - non-measured.

## 3. Discussion

The unique acute toxicities of CAR-T cell therapy, which can be fatal, require intensive monitoring and prompt management.^[1]^ Many CAR-T cell therapy patients will experience clinical features of ICANS. Although treatment guidelines exist for ICANS, there is little guidance on how to approach patients with rare or unusual cardiotoxicity or neurotoxicity presentations. Many factors probably affect the onset, peak level, duration, and type of acute toxicity that will occur after treatment with various CAR‐T cell products, and this variability should be considered when monitoring and treating each patient. Here we report a case of autonomic dysfunction POTS after CAR-T cell therapy following Axi-Cel for refractory PMBCL. The mechanism of CAR-T cell neurotoxicity remains to be well-elucidated, albeit several possible mechanisms of ICANS after CAR-T therapy have been described.^[4]^ The most widely accepted mechanism is driven by systemic inflammation and cytokine production, inducing systemic capillary leak and subsequent dysfunction of the BBB. This triggers a further local inflammatory response and CNS inflammation, resulting in abnormal neuronal functioning. Following CAR-T cell infusion, the most common initial ICANS symptoms include dysgraphia, word-finding difficulties, tremors, confusion, and somnolence. Hesitant speech and deterioration in handwriting are prominent and can rapidly progress to expressive aphasia and mutism. Furthermore, patients may develop a wide range of clinical manifestations like seizures, headaches, focal deficits, and even a decreased level of consciousness.^[5]^ ICANS usually overlap and correlate with CRS, although it has also been occasionally reported to occur independently from CRS.^[5]^ Previous works demonstrated that higher-grade CRS is associated with major adverse cardiovascular events.^[11]^ Cardiovascular side effects related to CAR-T therapy were described, including cardiovascular mortality, new onset heart failure, heart failure decompensation or new arrhythmias.^[7]^ In addition, hypotension is one of the main signs of CRS, thus, this finding raises the question regarding CRS role in orthostatic hypotension. A recent study provided evidence that patients with CRS who developed dysautonomia received treatment with steroids and tocilizumab, with favorable response, leading to CRS being unlikely to be a dominant cause of orthostatic hypotension.^[6]^ In our case, the CRS and later ICANS were solved when dysautonomia symptoms developed. Orthostatic hypotension was described as an effect of CAR-T therapy, but autonomic disorders are not clearly characterized as ICANS manifestation.^[6]^ POTS, which is one of the most common autonomic disorders, has a wide range of clinical manifestations, such as postural tachycardia, dizziness, orthostatic intolerance, presyncope, and exercise intolerance.^[7]^ There is a growing body of evidence that supports the theory that the ethology of POTS may have immune-mediated pathogenesis and its association with COVID-19 and ICANS was already reported.^[7]^ In our patient, the diagnosis of POTS was ruled out due to orthostatic hypotension associated with tachycardia and intolerance to orthostatism. The study of the autonomic nervous system revealed parasympathetic dysfunction, after resolution of the ICANS. Nowadays, the interest in understanding, diagnosing and treating dysautonomia growing as the COVID-19 pandemic caused millions of people worldwide being disabled with post-COVID syndrome, including post-COVID dysautonomia.^[12]^ In the COVID-19 series, patients were treated with non-pharmacologic therapies, and most required pharmacologic treatment for autonomic dysfunction and comorbid conditions and 8 months after COVID-19, 85% of patients had residual autonomic symptoms, with 60% unable to return to work.^[7]^ Recently, a case report associated severe dysautonomia as a manifestation of ICANS in a large B-cell lymphoma.^[3]^ Our patient presented PMBCL in progression when proposed to CAR-T therapy, after which developed early CRS and ICANS with rapid and progressive worsening altered mental status with global aphasia, with intermittent bursts of bilateral FIRDA in EEG and signs of mild encephalopathy, but while in resolution of ICANS and treatment reduction, dysautonomia occurred. It is described that a potential cause of orthostatic hypotension is medication. Certain classes of medications like anti-hypertensives, diuretics and neuroleptics are known to cause or exacerbate orthostatic hypotension.^[6]^ In our patient, adrenal insufficiency in the context of corticosteroid discontinuation was considered as differential diagnosis. However, the cortisol value and adrenocorticotropic hormone were in normal range reason why this hypothesis was discarded. Despite partial recovery of dysautonomia condition in this case, after 1 month, the patient maintained mild symptoms that affected daily activities, which demonstrates the disabling potential of this adverse effect. While the focus of CAR-T toxicity is primarily on CRS and ICANS, cardiac manifestations dysautonomic disorders (such as POTS and parasympathetic dysfunction) will be important to monitor in CAR-T patients. Although medications cannot cure autonomic dysfunction, can help relieving the symptoms. The decision to treat orthostatic hypotension should be based on the severity of orthostatic symptoms, the context in which they are occurring, the risk of recurrence, and the potential adverse effects of therapy. The therapeutic modalities of orthostatic hypotension include nonpharmacological and pharmacological options. Therapies of nonpharmacological nature include salt loading, head-of-bed elevation, orthostatic training programs and various physical counter maneuvres.^[13]^ Pharmacological agents used in the management of orthostatic hypotension belong to diverse groups and include drugs such as midodrine, fludrocortisone, β-blockers, octreotide, paroxetine and epoietin-α. The literature justifying their use is relatively small and difficult to interpret with regard to daily practice mainly because patient selection,.^[13,14]^ Our patient improved under nonpharmacological therapies, without needing pharmacological agents.

## 4. Conclusion

New-onset dysautonomic disorders (such as POTS and parasympathetic dysfunction) can occur as manifestations of ICANS in patients who experience persistent neurologic and cardiovascular symptoms after resolution of acute neurotoxicity. Further studies are needed to determine whether post-CAR-T autonomic disorders are established in autoimmunity and what type of antibodies or cytokines may be mediating the autoimmune and/or inflammatory process. Giving the relevance of this entity and its comorbidities, physicians should be aware that autonomic disorders may be a complication of CAR-T therapy and should consider appropriate diagnostic and therapeutic interventions in these patients. Increased awareness and effective monitoring of can help decrease the morbidity associated with these manifestations.

## Author contributions

**Conceptualization:** Dina Rochate, Antonio Manuel González-García.

**Data curation:** Dina Rochate.

**Investigation:** Dina Rochate, Carmen Santos Marcos.

**Supervision:** Estefanía Pérez-López, Monica Alaña, Lucía López Corral.

**Validation:** Lucía López Corral.

**Writing – original draft:** Dina Rochate, Antonio Manuel González-García.

**Writing – review & editing:** Carmen Santos Marcos, Estefanía Pérez-López, Monica Alaña, Lucía López Corral.
